# Functional and phylogenetic analyses of camel rumen microbiota associated with different lignocellulosic substrates

**DOI:** 10.1038/s41522-022-00309-9

**Published:** 2022-06-08

**Authors:** Javad Gharechahi, Sajjad Sarikhan, Jian-Lin Han, Xue-Zhi Ding, Ghasem Hosseini Salekdeh

**Affiliations:** 1 Human Genetics Research Center, Baqiyatallah University of Medical Sciences, Tehran, Iran; 2 Department of Systems Biology, Agricultural Biotechnology Research Institute of Iran, Agricultural Research, Education, and Extension Organization, Karaj, Iran; 3 Livestock Genetics Program, International Livestock Research Institute (ILRI), 00100 Nairobi, Kenya; 4 CAAS-ILRI Joint Laboratory on Livestock and Forage Genetic Resources, Institute of Animal Science, Chinese Academy of Agricultural Sciences (CAAS), 100193 Beijing, China; 5 Key Laboratory of Yak Breeding Engineering, Lanzhou Institute of Husbandry and Pharmaceutical Sciences, Chinese Academy of Agricultural Sciences (CAAS), 730050 Lanzhou, China; 6 Department of Molecular Sciences, Macquarie University, North Ryde, NSW Australia

**Keywords:** Metagenomics, Biofilms

## Abstract

Rumen microbiota facilitates nutrition through digestion of recalcitrant lignocellulosic substrates into energy-accessible nutrients and essential metabolites. Despite the high similarity in rumen microbiome structure, there might be distinct functional capabilities that enable different ruminant species to thrive on various lignocellulosic substrates as feed. Here, we applied genome-centric metagenomics to explore phylogenetic diversity, lignocellulose-degrading potential and fermentation metabolism of biofilm-forming microbiota colonizing 11 different plant substrates in the camel rumen. Diversity analysis revealed significant variations in the community of rumen microbiota colonizing different substrates in accordance with their varied physicochemical properties. Metagenome reconstruction recovered genome sequences of 590 bacterial isolates and one archaeal lineage belonging to 20 microbial phyla. A comparison to publicly available reference genomes and rumen metagenome-assembled genomes revealed that most isolates belonged to new species with no well-characterized representatives. We found that certain low abundant taxa, including members of Verrucomicrobiota, Planctomycetota and Fibrobacterota, possessed a disproportionately large number of carbohydrate active enzymes per Mb of genome, implying their high metabolic potential to contribute to the rumen function. In conclusion, we provided a detailed picture of the diversity and functional significance of rumen microbiota colonizing feeds of varying lignocellulose composition in the camel rumen. A detailed analysis of 591 metagenome-assembled genomes revealed a network of interconnected microbiota and highlighted the key roles of certain taxonomic clades in rumen function, including those with minimal genomes (e.g., Patescibacteria). The existence of a diverse array of gene clusters encoding for secondary metabolites unveiled the specific functions of these biomolecules in shaping community structure of rumen microbiota.

## Introduction

Domestic ruminants are the most important source of meat and dairy products for humans worldwide. They are characterized by a complex multi-chambered stomach, with the first and the most important compartment being the rumen^1^. The rumen provides suitable environmental conditions for housing dense and complex symbiotic microbial communities belonging to the three taxonomic domains of life^2^. Rumen microbiota play key roles in ruminant nutrition, health and productivity^3^. Without the aid of these microorganisms, ruminants are unable to digest and utilize plant-based polysaccharides as their sole nutrient sources. There is thus an obligate relationship between rumen microorganisms and their host since in addition to their role in feed digestion they are also linked to host physiology, rumen epithelium development and the regulation of host gene expression^4,5^. In addition, the composition of rumen microbiota has been linked to the animal’s feed digestion efficiency^6,7^, an economically important trait that is subject of many ruminant breeding programs. A recent large-scale analysis of rumen microbiome of over 1000 cows suggested that there is a heritable core microbiome that is linked to host phenotypes such as productivity and methane emission^7^. Based on this finding, they argued that this core microbiome should only be engineered at very early age of colonization if one sought to utilize microbiome engineering in breeding programs^7^.

Within the rumen microbiome, bacteria are the most abundant and functionally important microorganisms, having a significant contribution to lignocellulose degradation. Colonization of feed particulates by rumen microbiota initiate shortly after their entry into the rumen and bacteria are the first group that adhere^8,9^. Bacteria that form a dense biofilm on the feed surface play central role in lignocellulose digestion^10,11^. The cooperation between taxonomically and metabolically diverse bacteria in the biofilm greatly enhances lignocellulose breakdown in the rumen^12^. Forage physicochemical properties are among factors directly affecting rumen microbial colonization and digestion^8,13–19^. Our previous study on rumen microbiota in cattle, for example, revealed that lignocellulose-degrading Fibrobacteria tended to populate forages with high neutral detergent fiber while *Ruminococcus* outcompeted other lineages in colonization of forages with low acid detergent lignin^13^. These findings suggested that rumen bacteria have evolved to populate on feeds of varying chemical properties, but their differential colonization highlighted that forage chemical compositions also contributed to the extent of microbial colonization and digestion in the rumen.

In light of advances made in bioinformatic analysis of metagenome sequences, it has now become possible to recover draft genome sequences of rumen bacteria and archaea particularly that of those escaping in vitro culture conditions. This has enabled us to explore metabolic potential of individual species of rumen microbiota and their synergistic interaction during lignocellulose degradation and fermentation. Compared to cattle rumen microbiota which has been widely explored using metagenome sequencing, there is limited knowledge about microbial community inhabiting camel rumen and their diversity and metabolic capabilities with respect to lignocellulose degradation and fermentation.

Dromedary camels (*Camelus dromedarius*) live in deserts and dry lands in north and northeastern parts of Africa through the Middle East countries up to India. These animals have naturally adapted to harsh environmental conditions, with the ability to withstand hot climate and extreme water deprivation, for instance, they can survive for up to 40 days without drinking. They mostly browse on shrubs and woody plants with complex lignocellulosic structures and high content of anti-nutritional components, including tannins, saponins, phytates, oxalates, and phenolic compounds, which make them typically inedible for other herbivorous animals. Although they ruminate like other ruminant animals such as cattle, sheep and goat, they are known as pseudo-ruminants and thus classified in a separate family of ruminants^20^. In contrast to the true ruminants which have a four-chambered stomach (rumen, reticulum, omasum and abomasum), camels have a three-chambered stomach consisting of C1 or rumen, C2 and C3 compartments with a no well-differentiated omasum-like compartment. Given the complexity and the recalcitrant nature of plant residues constituting camel’s diet and the ability of its rumen microbiota to utilize such rough plant materials, we selected this animal for an in situ rumen incubation of different lignocellulosic substrates followed by whole metagenome sequencing of the attached microbiota. Previous metagenome analysis of rumen microbiota of dromedary (single-humped) and Bactrian (two-humped) camels revealed almost similar microbial compositions compared to other ruminants at least at higher taxonomic ranks^21–24^. However, due to their unique forestomach anatomy, extreme living conditions and recalcitrant nature of their diet, it is expected that their rumen microbial communities be functionally distinct from those of true ruminants. We aimed to taxonomically identify and functionally characterize taxa involved in the breakdown of forages of varying lignocellulosic properties in the camel rumen. We hypothesized that variations in the lignocellulose compositions of the incubated biomasses will enable us to capture as much as rumen microbial diversity in substrate attachment and utilization as possible. We also considered whether the camel rumen microbial communities and their metabolic functions are distinct compared to other ruminant species. Using 490 Gbp of metagenome data, we reconstructed the genome sequences of 590 bacterial isolates and one archaeal lineage with diverse metabolic and carbohydrate-degrading capabilities and further inferred their potential contribution to lignocellulose degradation and fermentation in the camel rumen.

## Results

### Metagenome sequencing and assembly

**Fig. 1:**
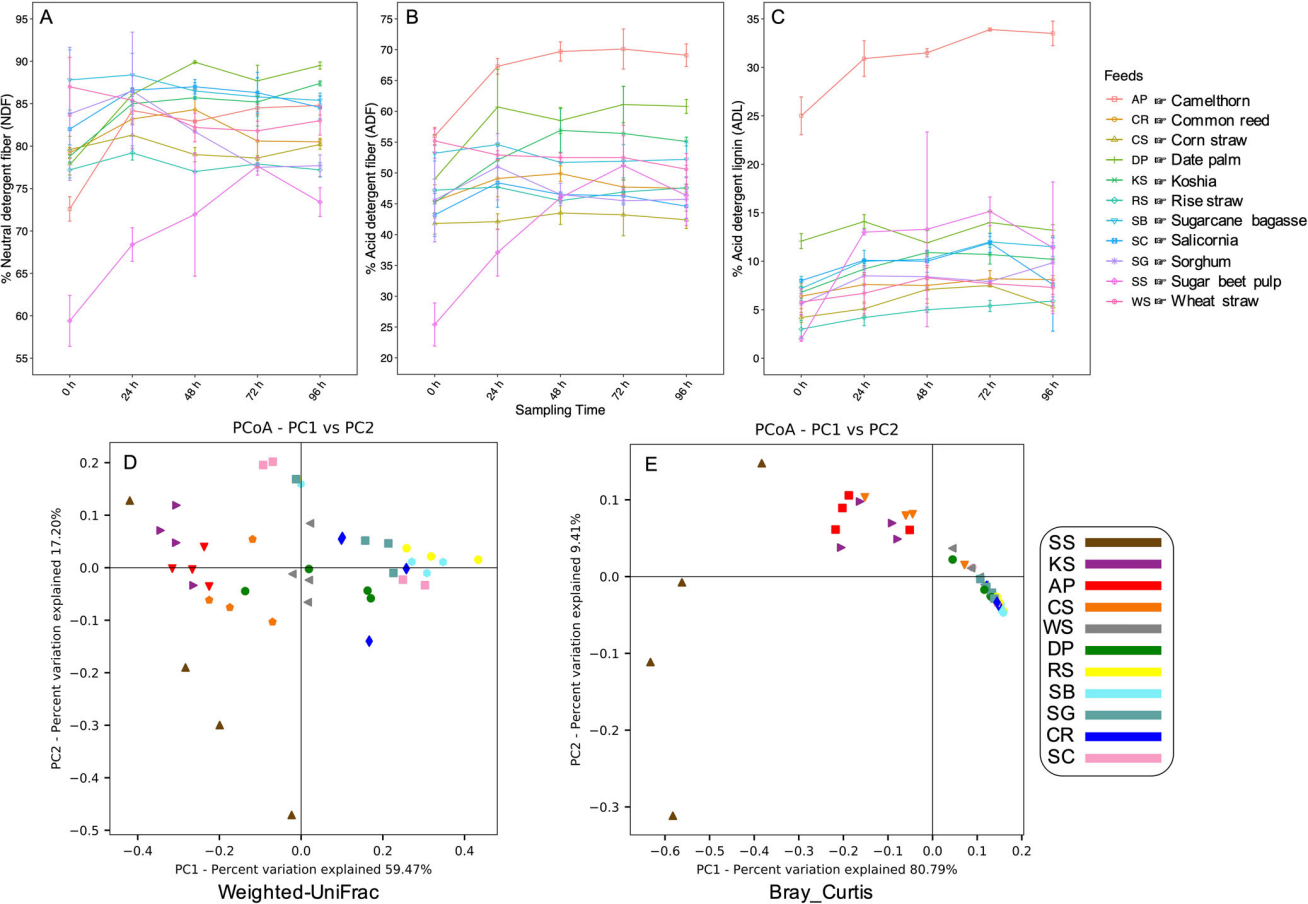
Forage physicochemical properties and the diversity of their attached microbiota during incubation in the camel rumen. Forages degradability was evaluated by measuring (**A**) neutral detergent fiber (NDF), (**B**) acid detergent fiber (ADF) and (**C**) acid detergent lignin (ADL) concentrations during incubation in the rumen. Concentrations are presented based on percentage of dry matter. Data points are mean and error bars represent standard errors. Beta diversity of camel rumen microbiota attached to the forages was explored by searching reads against ~1.1 M lineage-specific marker genes using MetaPhlAn^83^. Principal coordinate analysis (PCoA) plot shows community diversity of rumen microbiota attached to 11 different forages incubated in the camel rumen based on (**D**) weighted UniFrac and (**E**) Bray_Curtis dissimilarity matrices. Samples are grouped based on feeds and each feed is represented by a unique shape and color.

To investigate the dynamic changes in the colonization cycle and the extent of substrate specialization of rumen microbiota, different lignocellulosic biomasses were in situ incubated in the rumen of two body-weight and age-matched rumen-fistulated dromedary camels for a period of 96 h with 24 h sampling intervals. Eleven lignocellulosic substrates, including straws of wheat (WS), corn (CS), rice (RS), common reed (CR), koshia (KS), date palm (DP), sorghum (SG), salicornia (SC), camelthorn (AP), sugar beet pulp (SS), and sugarcane bagasse (SB) were selected for in situ rumen incubation. Figure 1 shows NDF, ADF and ADL concentrations of the plant biomasses and their degradability during incubation in the rumen. As a total measure of forage digestibility, NDF concentration varied between feeds with DP and SB having the highest and SS having the lowest concentrations (Fig. 1A). The incubated forages also varied with respect to the content of ADF and ADL with AP and DP having the highest and CS, SS, and having RS the lowest concentrations (Fig. 1B, C).

The whole metagenome sequencing of rumen microbiota tightly attached to the 11 substrates at four sampling intervals (24 h, 48 h, 72 h and 96 h) yielded up to 490 Gbp of sequencing data. A combined assembly of all sequences produced 2,727,608 contigs with minimum length 1 Kbp, maximum length 585 Kbp and an N50 of 4.7 Kbp. A combined coverage and tetranucleotide based binning of contigs ≥ 2 Kbp resulted in 1690 genome bins. A two-stage pair-wise comparison of genomes at 99% and 90% ANIs resulted in 590 bacterial isolates and one archaeal lineage (hereafter called rumen uncultured isolates, RUIs) belonging to 20 microbial phyla with completeness ≥75% and contamination ≤10% (Supplementary Data 1). Among these RUIs, 230 had a completeness ≥90% and contamination ≤5%, of which only two had a full complement of rRNA genes (23S, 16S and 5S rRNAs) and at least 18 standard tRNAs, and thus could be considered as high-quality draft MAGs based on the criteria defined by the Genomic Standards Consortium^25^. Even though that the number of the RUIs with completeness >90%, contamination <5% and >18 tRNAs was relatively high (133 RUIs), but the difficulty in assembling rRNA genes from data significantly reduced the number of high-quality RUIs. The RUIs had genome sizes between 0.71 and 5.23 Mbp and N50 values from 5 to 287 Kbp. The smallest RUI was a Saccharimonadaceae bacterium (RUI400) while the largest one was a Lentisphaeria bacterium (RUI478). Generally, Lentisphaeria (RUI186 and RUI478) and Planctomycetes (RUI074 and RUI165) had the largest (4.9 and 3.4 Mbp, respectively) while Saccharimonadia (4 RUIs) and Alphaproteobacteria (6 RUIs) had the smallest (0.88 and 0.98 Mbp, respectively) average genome sizes.

### Taxonomic classification of the whole metagenomes and RUIs

Taxonomic classification of metagenome sequences using clade-specific marker genes revealed that on average 91% of the sequences were distantly related to the existing reference genomes. Among the sequences with known taxonomic origins, 5.3% were associated with Bacteroidota, 3% with Firmicutes, 0.17% with Fibrobacterota and 0.07% with Spirochaetota, followed by Actinobacteriota, Synergistota and Proteobacteria which collectively accounted for 0.05% of the sequences. Weighted UniFrac dissimilarity matrix showed that differences in community compositions across feeds were sufficient for their separation (Fig. 1D). However, Bray_Curtis diversity matrix could not differentiate between most samples with only SS, KS, AP and CS to be well-separated (Fig. 1E). This finding suggested that differences in community structures across substrates were likely driven by taxonomic variations. Both indices revealed distinct community structure for SS (sugar beet pulps) samples. Particularly, the proportions of known taxa were significantly higher in this substrate (average 42% vs 5%). The easy availability of carbohydrates (mostly glucans) in this substrate presumably enhanced the colonization of fast-growing *Prevotella* and *Streptococcus*. A high abundance of *Streptococcus* is observed when animals are offered concentrate or starch-rich diets.

The reconstructed RUIs were taxonomically classified based on a concatenated protein-based phylogeny representing >190,000 prokaryote genomes using GTDB-Tk^26^. Over 56% of the RUIs were assigned to the phylum Firmicutes_A (332), 20% to Bacteroidota (119), 7% to Firmicutes (46) and 6% to Spirochaetota (38), and these four phyla collectively accounted for greater than 90% of all RUIs (535). The remaining 56 RUIs were assigned to 16 different phyla, including Proteobacteria (9), Firmicutes_C (7), Verrucomicrobiota (6), Desulfobacterota_A (5), Actinobacteriota (5), Patescibacteria (5), Cyanobacteria (3), Synergistota (3), Elusimicrobiota (3), Fibrobacterota (2), Planctomycetota (2), Eremiobacterota (2), Euryarchaeota (1), Desulfuromonadota (1), Campylobacterota (1) and Firmicutes_B (1). Details of inferred taxonomies are presented in Supplementary Data 2. Over 85% of the RUIs were not reliably assigned to the existing species, suggesting that most uncultured microbial species in the camel rumen lacked representative candidates in reference databases. Further taxonomic classification using PhyloPhlAn also revealed that over 78% of the RUIs were not closely related to the known species-level genome bins (SGBs) as defined by Pasolli, et al^27^., further highlighting their novel taxonomic origins.

**Fig. 2:**
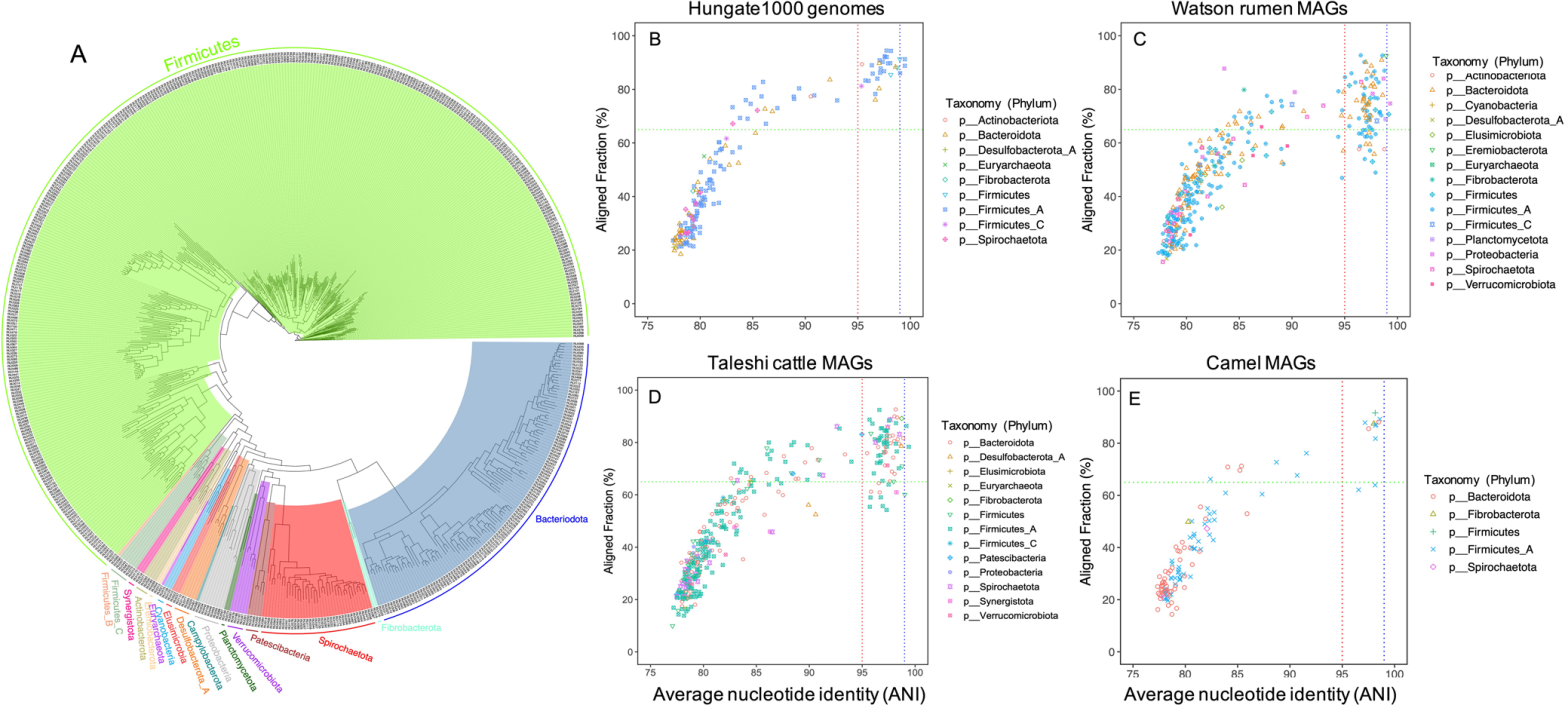
Taxonomic classification of RUIs and their sequence comparison with previously reported rumen cultured isolates and metagenome-assembled genomes (MAGs). **A** The tree shows phylogenetic relationship of 591 RUIs taxonomically classified at the phylum level. Taxonomies were assigned based on comparison with ~40,000 reference genomes from Genome Taxonomy Database (GTDB) using GTDB-Tk^26^. Average nucleotide identities (ANIs) along with aligned fractions (AFs) of the RUIs against rumen cultured isolates (*n* = 408) sequenced by Hungate1000 genome project (**B**), rumen metagenome-assembled genome (MAGs) from Scottish cattle (*n* = 4941) (**C**), Taleshi cattle (*n* = 538) (**D**) and camel (*n* = 65) (**E**). An AF of 65% was considered as cutoff for correct species delineation.

Based on the GTDB taxonomies, 101 RUIs were remained unannotated at the genus level, seven at the family level and one at the order level (Supplementary Data 2). The majority of the RUIs unassigned at the genus level belonged to the families Lachnospiraceae (*n* = 45), Anaerovoracaceae (*n* = 6), CAG-826 (*n* = 5), Oscillospiraceae (*n* = 4), Treponemataceae (*n* = 4) and CAG-272 (*n* = 4), most of which are known as core members of the animal GIT microbial communities making a significant contribution to lignocellulose degradation in the rumen. Figure 2A shows the phylogenetic tree of the 591 RUIs reconstituted from camel rumen. Clades are colored according to the phylum-level taxonomies inferred by GTDB-Tk (Supplementary Data 2).

To get further insight into taxonomic novelty of the RUIs, we compared our RUIs with a set of rumen cultured representatives from the Hungate1000 genome project *n* = 408^28^, cow rumen MAGs *n* = 4941^29^ and our previously reconstructed rumen MAGs from Iranian native Taleshi cattle *n* = 538^19^ and single-humped camel *n* = 65^30^ by calculating their pair-wise ANIs (Fig. 2B–E). Considering a minimum alignment fraction (AF) 65% which is required for a correct species delineation^31^, only 30 RUIs had >95% ANIs with the cultured isolates from Hungate1000 genome project, 66 with the cow rumen MAGs, 69 with the Taleshi cattle MAGs and nine with the camel MAGs, suggesting their shared species-level taxonomies. A relatively high representation of RUIs in our previously reconstructed MAGs (12.8% in Taleshi cattle and 13.8% in camel’s MAGs) suggested a shared geographical origin for most rumen microbiota regardless of their different host species. Collectively, only 143 camel RUIs had >95% ANIs over 65% of AF with genomes from these four datasets. Most of these RUIs belonged to the families Lachnospiraceae (*n* = 55), Bacteroidaceae (*n* = 14), UBA932 (*n* = 12) and Ruminococcaceae (*n* = 8), which are mostly known as core members of the rumen microbes shared across different ruminant species^21,32^.

### Community diversity and differential analysis of the RUIs

**Fig. 3:**
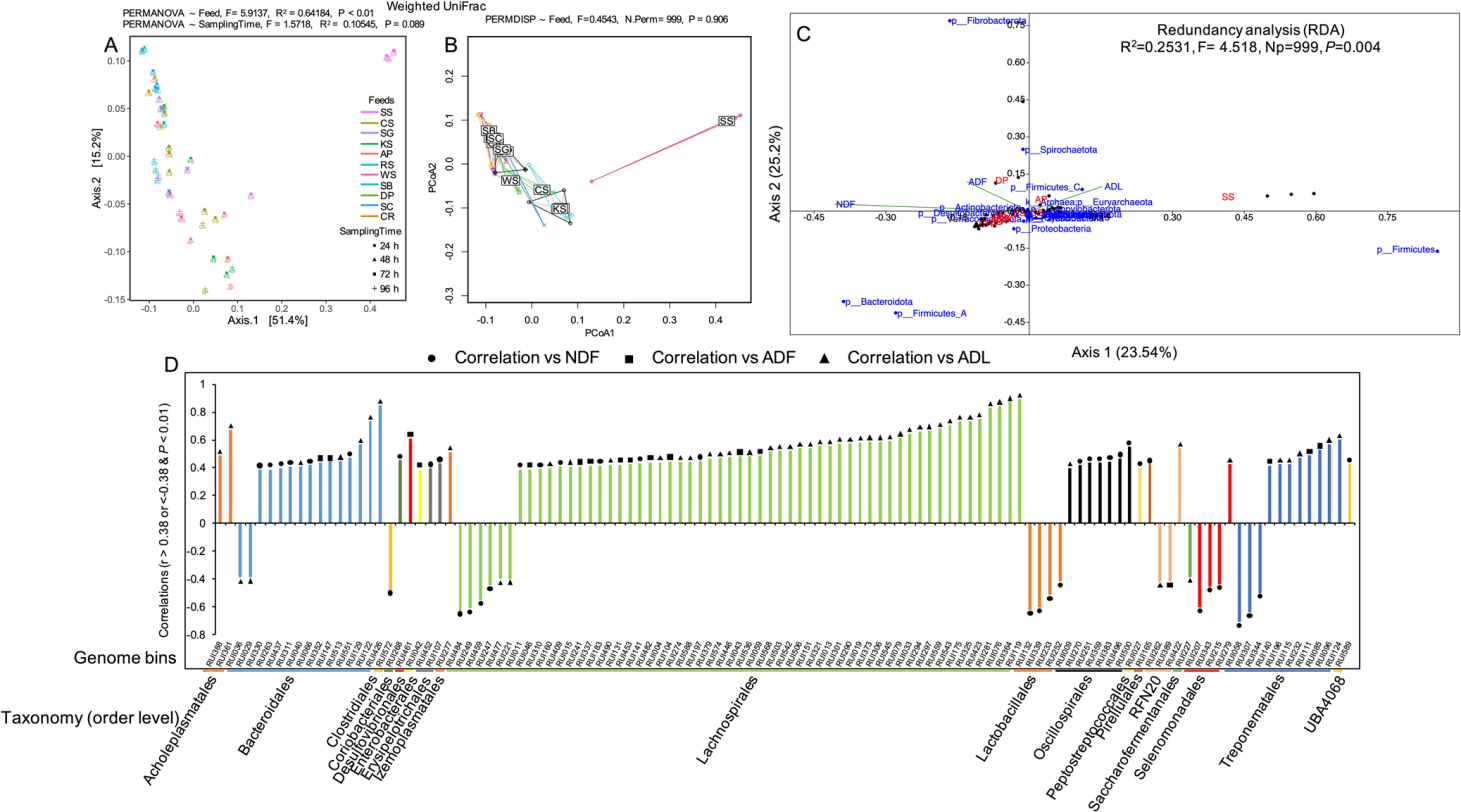
Beta diversity analysis of the rumen uncultured isolates (RUIs) reconstituted from metagenome-assembled contigs. **A** PCoA plot showing significant separation of forages in a two-dimensional space based on community diversity (weighted UniFrac dissimilarity matrix) of RUIs reconstituted from metagenomes of forage-attached microbiota. Each feed is represented by a unique shape and color. Significant differences in community compositions were tested using permutation multivariate analysis of variance (PERMANOVA). **B** The homogeneity of group dispersion between forages was also tested using permutational multivariate analysis of dispersion (PERMDISP). **C** RUIs abundance profiles (phylum level) were correlated with forage degradation indices (i.e., NDF, ADF and ADL) using redundancy analysis (RDA). The first two axis explaining the highest variations are displayed. RDA biplot was created using PAST software^95^. **D** Significant correlations of genome bin abundances (grouped at taxonomic level of order) with forage physicochemical properties. Correlations with *r* > 0.38 or *r* < −0.38 and *P*-value < 0.01 were considered statistically significant.

The abundance of RUIs was quantified by aligning reads back to the genomes using quant_bins module from MetaWRAP pipeline^33^. Heatmap clustering (Fig. S1A) revealed similar and shared microbial communities attached to WS, CS, KS, SS and AP substrates. Time scale separation was also apparent in certain biomasses at early time points, i.e., 24, 48 and 72 h. This result demonstrated the dynamic changes in abundance and colonization succession of rumen microbial communities during lignocellulose fermentation in the rumen regardless of the chemical properties of the substrates. Community diversity analysis using weighted UniFrac dissimilarity matrix (Fig. 3A, B), which considers both abundance and phylogeny of the genomes, showed significant differences in community compositions among substrates (PERMANOVA, *P* < 0.01) but not across sampling intervals (PERMANOVA, *P* > 0.05), a pattern being further confirmed by dispersion analysis (PERMDISP, *P* < 0.05). This finding signified that differential colonization by taxa of varying taxonomic origins was likely critical for efficient degradation of different lignocellulosic substrates in the rumen. Correlating taxa abundances with overall forage physicochemical properties showed higher colonization of cellulose-rich substrates, including AP and DP with Fibrobacterota species with high cellulose-degrading potential (Fig. 3C). Higher attachment of Bacteroidota and Firmicutes_A to substrates with increased NDF concentrations, including SB and SC was also noticeable (Fig. 3C, Fig. S1B). Correlating RUIs abundances and physicochemical properties of forages (i.e., NDF, ADF and ADL) revealed significant positive correlations (*r* > 0.38 and *P* < 0.01) for 13 Bacreriodales (out of 119), 51 Lachnospirales (out of 220), 7 Oscillospirales (out of 55) and 8 Treponematales (out of 34) genomes (Fig. 3D). A negative correlation (*r* < −0.4 and *P* < 0.01) was also noted for 4 Lactobacillales (out of 6) and 3 Selenomonadales (out of 7) with NDF concentrations of feeds during rumen incubation.

Differential analysis of the RUIs during substrate incubation revealed the higher abundance of genomes belonging to Firmicutes_B, Proteobacteria and Desulfuromonadota phyla during the initial hours of rumen incubation and a higher abundance of Spirochaetota at the later time points (ANCOM, Bonferroni corrected *P* < 0.05).

### Functional annotation of the whole metagenomes of fiber-attached microbiota

Prediction of ORFs in assembled contigs resulted in 20,909,730 sequences ≥ 50 aa with an average length of 198 aa. COG classification revealed that over 10% of the sequences were associated with translation, ribosomal structure and biogenesis category (COG category J), 9.2% with carbohydrate transport and metabolism (G), 7.56% with amino acid transport and metabolism (E), 7% with cell-wall/membrane/envelope/biogenesis (M), 6.99% with replication and recombination repair (L), 6.85% with general function prediction only (R), 5.6% with energy production and conversion (C) and 4% with transcription (K). Sequence similarity analysis revealed that 83% and 68% of the sequences aligned with an e-value cutoff 1e−5 to at least one hit in the nr and env_nr databases, respectively. Only 409,647 sequences were 100% identical with the proteins in nr and 4015 with those in env_nr databases. In addition, greater than 85% of sequences shared less than 90% identity with entries in nr and 99% with those in env_nr databases, indicating their novelty.

Annotation with KEGG orthologs (KOs) resulted in 4,249,411 hits (20%), which were mapped to 7,112 unique KO terms, 408 KEGG pathways, 147 KEGG modules and 47 Brite functional hierarchies. However, of the proteins encoded by RUIs, 613,246 (46.6% of the proteomes) were annotated to 4601 KO terms in the KEGG database. The higher annotation rate for RUI ORFs compared to the whole metagenomes was likely due to the higher average ORF size in the RUIs (336 vs. 198 aa) and thus the higher number of full-length genes. Comparison of the number of unique KO terms identified in our RUIs with those identified in MAGs (*n* = 1200) from the African cattle rumen microbiome^34^ showed higher KO terms in our RUIs (4601 vs. 4000). Our camel RUIs contained 1403 KO terms which were absent in the African cattle rumen MAGs and they were mostly associated with metabolism (mostly enzymes (727)), genetic information processing (mostly transcription factors (68)) and signaling and cellular processes (mostly transporters (242), secretion system (68) and prokaryotic defense system (64)). We found complete modules for antibiotic (beta-Lactam resistance, M00627) and multidrug (efflux pump MepA, M00705) resistance in the camel RUIs, which were absent in the African cattle rumen MAGs. Nonetheless, most of the 800 GOs present in the African cattle rumen MAGs, but absent in our camel RUIs were associated with metabolism (mostly enzymes (476)), genetic information processing (mostly transcription factors (49)) and signaling and cellular processes (mostly two-component system (69) and bacterial toxins (10)).

Out of 4601 KO terms identified in RUIs, 53% (2448 KO terms) were affiliated to 315 KO pathways. Supplementary Data 3a shows the list of KO terms identified in RUIs and the abundance profile of KOs affiliated to 76 major KEGG pathways. Most of these KO terms were associated with metabolic pathways (1408 KO terms), biosynthesis of secondary metabolites (480), microbial metabolism in diverse environments (404), ABC transporters (233), biosynthesis of cofactors (231), carbon metabolism (201), two-component system (180), biosynthesis of amino acids (177) and methane metabolism (116). A gene enrichment analysis revealed the higher abundance of genes associated to metabolic pathways, microbial metabolism in diverse environments, amino-acyl tRNA biosynthesis and biosynthesis of secondary metabolites in AP compared to RS, the forages with the highest and the lowest ADL concentrations, respectively, and in SS compared to SB the two forages with the highest differences in NDF concentrations (Fig. 1C). No specific gene enrichment was detected in comparisons of other forages consistent with their limited differences in physicochemical characteristics (Fig. 1).

### Identification of carbohydrate active enzymes (CAZymes)

CAZymes were predicted in both the whole metagenomes and the RUIs using dbCAN database with an e-value cutoff 1e-15 and a minimum query coverage of 35%. Among more than 20 million protein-coding sequences predicted in assembled contigs, 526,935 (2.5%) were found to encode for CAZymes. Of which, ~49.5% encoded for glycoside hydrolases (GHs), 30.8% for glycoside transferases (GTs), 10.7% for carbohydrate esterases (CEs), 6.3% for carbohydrate binding modules (CBMs), 1.9% for polysaccharide lyases (PLs), 0.59% for auxiliary activity (AAs) domains, 0.1% for S-layer homology domains (SLH) and 0.02% for cohesin. The absence of independent dockerin domain could be because this domain is always appended to other CAZyme catalytic domains, hence proteins containing this domain were annotated based on their catalytic domains.

**Fig. 4:**
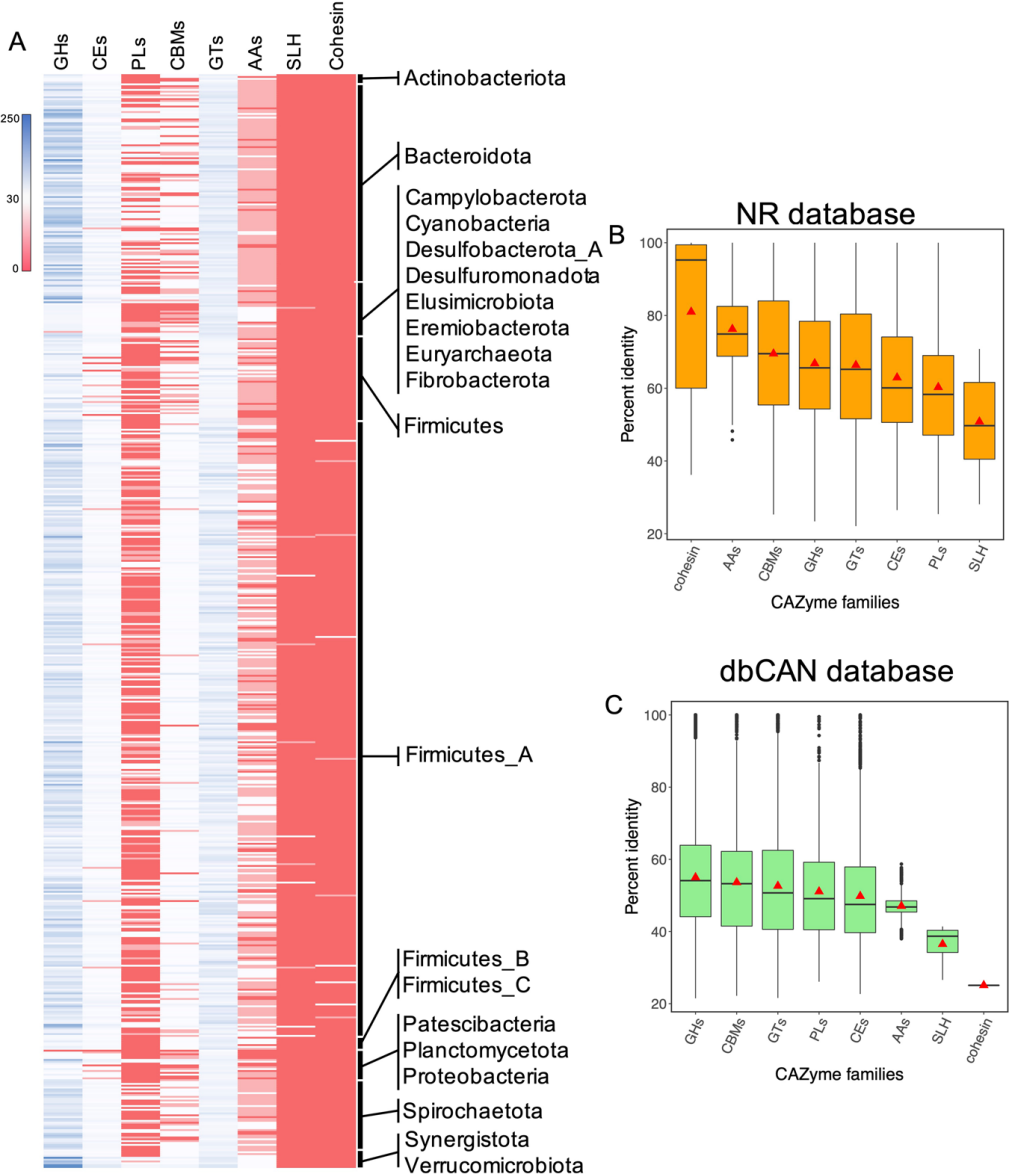
The profile of carbohydrate active enzymes (CAZymes) predicted in the RUIs and their sequence comparison with public databases. Heatmap shows the abundance profile of CAZymes in RUIs classified at the phylum level (**A**). The sequences of the predicted CAZymes were searched against non-redundant (nr) protein database (**B**) and dbCAN database (**C**). GHs, glycoside hydrolases; GTs, glycosyl transferases; PLs, polysaccharide lyases; CEs, carbohydrate esterases; AAs, auxiliary activities; and CBs, carbohydrate binding modules. The boxes represent the median (center line) and interquartile ranges, red triangle represents mean and whiskers extend to one and a half times of the interquartile range.

The prediction of protein-coding genes in the RUIs yielded 1,314,286 sequences, of which 61,141 (4.65%) were annotated as CAZymes. Around 52% of the CAZymes were annotated as GHs, 29% as GTs, 10.2% as CEs, 4.7% as CBMs, 1.7% as PLs, 1.12% as AAs, 0.04% as SLH and 0.03% as cohesin (Supplementary Data 4). The abundance profiles of CAZymes in the RUIs classified at the phylum level are depicted in Fig. 4A. There was no significant deviation in the abundance profiles of camel CAZymes from those identified in Taleshi cattle^19^, African cattle^34^ and Scottish cattle rumen MAGs^29^, suggesting a similar degradation potential for rumen microbiota across different host species.

Normalizing the number of GHs predicted in each RUI to its genome size revealed the highest average density of GHs in the RUIs affiliated to Verrucomicrobiota (37.9 GH/Mb), followed by Bacteroidota (30.6), Fibrobacterota (28.7), Planctomycetota (28.4), Spirochaetota (20.8) and Firmicutes_A (19.5). CEs were more densely represented in Planctomycetota, Fibrobacterota, Bacteroidota, and Verrucomicrobiota with an average 6 CEs/Mb of their genomes (Fig. S2). PLs, however, were more enriched in Fibrobacterota (4.7 PLs/Mb), Verrucomicrobiota (2.15) and Bacteroidota (1.62). Cohesin domain containing proteins were only detected in a few genome bins of Firmicutes_A phylum, including members of the families Ruminococcaceae (7 RUIs), Acutalibacteraceae (1), Lachnospiraceae (1) and Clostridiaceae (1).

To investigate the extent of sequence novelty, the predicted CAZymes were BLASTP searched against the nr protein database. The predicted sequences shared an average of 66% identity with the proteins in this database. Only 545 proteins were completely identical to the proteins in the nr database and 36,071 (59%) had an identity less than 70%, suggesting a high sequence novelty. Cohesin domain containing proteins showed the highest (80%) while SLH domains the lowest (50%) average identities with the proteins in the nr database. The average sequence identities were significantly dropped when the CAZyme sequences were compared with the dbCAN database, among which GHs had the highest average identities (55%) and cohesins the lowest (25%). The low sequence identity of cohesin domains could be attributed to the low representation of this domain in dbCAN compared with the nr databases or the high novelty of our predicted sequences (Fig. 4B, C). Cohesins are usually detected in arrays of tandemly repeated domains in scaffoldin proteins, key components of cellulosome complexes^35^. Four RUIs contained proteins with such configuration (2–4 cohesin repeats), including three affiliated to *Ruminococcus flavefaciens* (RUI014, RUI512 and RUI532) and one to *Clostridium* sp. (RUI572), indicating their apparent capabilities for cellulosome complex production.

CAZymes are frequently modular proteins, in which catalytic domains are associated with other non-catalytic or catalytic domains, a characteristic that improves their targeted and/or concerted action during complex carbohydrate breakdown^36^. We found that 4660 CAZymes in 552 RUIs had two or more CAZyme domains. The majority of these modular CAZymes were detected in cellulolytic bacteria, including genera *Ruminococcus* and *Fibrobacter*. In particular, ~39% of all CAZymes predicted in RUI009 and RUI259 (both *R. albus*) and 31% of CAZymes in RUI512 (*R. flavefaciens*) were multidomain proteins. Around 58% of all CBM48 domains predicted in the RUIs (430 sequences) were appended to the GH13_9 module and 90% of CBM67 domains (331) were associated with the GH78 α-L-rhamnosidase catalytic domain. Up to 79% of the cellulose binding module CBM4 were appended to GH9 endoglucanase. In addition to GH9, CBM4 was also widely associated with GH16, GH10, GH148, GH5_55 and GH128 endoglucanases and xylanases as well as CE1, CE2 and CE4 xylan esterases, suggesting a multi substrate binding capacity for this domain and its key role in lignocellulose degradation in the rumen. Starch binding domain CBM34 was also frequently (42%) associated with GH13_39 α-amylase. Details of multidomain CAZymes are presented in Supplementary Data 5.

To get access to the complex lignocellulosic substrates, carbohydrate active enzymes must be either secreted into the extracellular milieu or delivered into the periplasmic space. A search for the presence of secretion signals in CAZymes predicted in the RUIs revealed that only 23% were secreted or periplasmic (Supplementary Data 6). Analysis of the taxonomic origins of CAZymes with secretion signals revealed that only certain taxonomic groups have made a significant contribution to secretory CAZymes and thus to lignocellulose degradation in the rumen, including members of Planctomycetota (60% of their CAZyme repertoire being secreted), Bacteroidota (48%), Fibrobacterota (47%), Verrucomicrobiota (36%) and Firmicutes_B (20%). The proportions of secretory CAZymes in different taxa were similar to that in the Taleshi cattle rumen microbiome^19^. Only in certain RUIs, including members of the genera RC9 gut group (RUI130, RUI393, RUI533, RUI535, RUI375 and RUI547), *Prevotella* (RUI435, RUI579, RUI067, RUI122 and RUI080) and family Thermoguttaceae (RUI074), greater than 60% of their CAZymes were predicted to be secretory proteins.

To examine the association of CAZyme families with specific substrates, the abundance of the RUIs encoded CAZymes were traced in metagenome of microbiota associated to different substrates. This analysis revealed that certain CAZymes, including members of GH2, GH3, GH94, and GH31 glucosidases and GH51 and GH9 endogluconases and GH10 endo-1,4-β-xylanases were significantly more enriched in microbiota associated to AP, the forage with the highest ADF and ADL concentrations compared to RS with low ADF and ADL contents. An interesting finding was the high representation of SLH domains in CR, the forage with high NDF concentration. SLH domains are responsible for non-covalent anchorage of extracellular proteins to the cell surface, particularly CAZymes and cellulosomal complexes^35^. An increased representation of GH92 mannosidases, GH78 rhamnosidases, xylan binding domain CBM37 and galactan binding domain CBM61 was also noted in RS microbiota.

### Prediction of polysaccharide utilization loci (PULs) in Bacteroidota genomes

Out of 119 RUIs taxonomically affiliated to Bacteroidota, 49 belonged to the family Bacteroidaceae, nine to Paludibacteraceae and one to Marinilabiliaceae while the remaining ones were closely related to uncultured bacteria in UBA groups, including P3 and F082 (Supplementary Data 7). In Bacteroidota, CAZymes are organized into specific gene clusters termed PULs, featured by the presence of a pair of SusC/D genes, which encode sugar transporters. This special gene organization enables the bacterium to recognize, import and degrade specific glycan substrates^37^. The composition of enzymes in PUL gene clusters determines the lignocellulose-degrading potential of the bacterium and the specificity of substrates it can degrade^38^. A search for the PULs in Bacteroidota genomes revealed the existence of 861 PUL-like structures in 95 genomes (Supplementary Data 7). The majority of these PULs (74 %) were detected in the members of the genus *Prevotella* (~40 %) and RC9 gut group (~34%), further suggesting their significant contribution to polysaccharide degradation in the camel rumen. Previous analysis of moose rumen metagenome also pointed that more than half of the expressed PULs originated from these two taxonomic groups, many of which were found to encode for hemicellulose and pectin degrading enzymes^39^. Members of RC9 group bacteria are uncultured gut bacteria belonging to the family Rikenellaceae, which are characterized by their small genome sizes and capabilities to produce propionate, acetate and/or succinate^28,40^. The average GH density in RC9 genomes was around 41 per Mb, which was significantly higher than that of the entire phylum (~30 GHs/Mb). This finding along with the high abundance of secretory CAZymes in these taxa indicated their significant roles in lignocellulose degradation in the camel rumen.

Around 48% of the predicted PULs lacked any known CAZyme in their vicinity, a pattern consistent with findings in cattle^19,41^ and other ruminants^38,39^. A search for CAZymes frequently presented in the PULs revealed that only certain GH families, including members of GH3 β-glucosidases/xylan 1,4-β-xylosidases, GH2 β-galactosidases/β-mannosidases, GH92 mannosyl-oligosaccharide α-1,2-mannosidases, GH26 β-mannanases, GH28 polygalacturonases and GH97 glucoamylases/α-glucosidases, were more frequently occurred in the PULs. Among other CAZymes, CE1 acetyl xylan esterases, CE10 arylesterases, CE8 pectin methylesterases and PL1_2 pectate lyases were also more highly represented in the PULs. Consistent with our finding in the cattle rumen^19^, the low representation of cellulose-degrading enzymes, including members of the families GH5, GH9, and GH44 in PULs, indicated that PULs make a minor contribution to the crystalline cellulose degradation^38^. However, the high representation of pectin and xylan degrading enzymes, including members of GH2, GH3, GH26, GH28, GH78 and GH97 along with CE1, CE8 and PL1_2, indicated that PULs have been evolved for targeted degradation of heterogenous lignocellulosic substrates, such as xylans, xyloglucans and pectins, which require a concerted action of multiple different CAZymes.

### Metabolic characterization of the RUIs

**Fig. 5:**
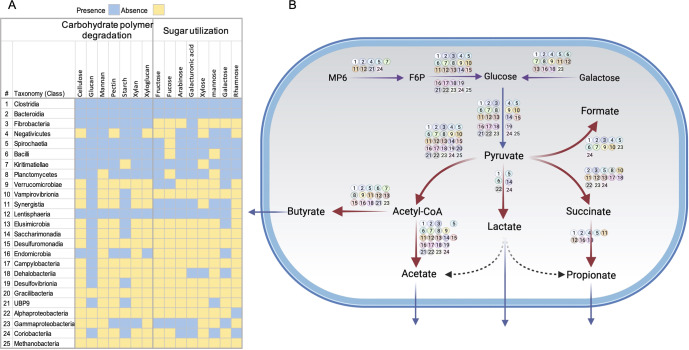
Metabolic characterization of RUIs. RUIs were characterized with respect to their abilities for major polysaccharides degradation, sugar utilization and fermentation. RUIs were grouped into class level taxonomic clades based on taxonomies inferred by GTDB-Tk. Metabolic pathways were reconstructed based on the presence of key marker genes of each metabolic pathway using KOfam annotations. The details of Hidden Marcov Model (HMM) profiles searched are presented in Supplementary Data 3.

Given the high phylogenetic diversity of the reconstructed RUIs, we sought to infer their potential contribution to carbohydrate fermentation in the camel rumen by assigning metabolic functions to the proteins predicted in individual genomes. The ability to metabolize carbohydrate monomers is determined by the presence of kinase, isomerase and aldolase enzymes responsible for the conversion of hexoses and pentose sugars to metabolizable forms. Rumen symbiotic microorganisms provide the energy requirement of the host with energy-accessible volatile fatty acids (VFAs) such as butyrate, acetate and propionate through fermentation of sugars released during lignocellulose degradation^42^. To explore the potential of RUIs for carbohydrate utilization and fermentation, proteins encoded by individual genomes were annotated against KEGG modules for major metabolic pathways, including glycolysis, pentose phosphate pathway (PPP), TCA cycle and fermentation (Fig. 5, Supplementary Data 3b). RUIs widely encoded the components of glycolysis and PPP pathways except for the members of Patescibacteria which lacked genes for PPP and also certain genes of the glycolysis pathway, including glucokinase (*glk*), phosphofructokinase (*pfk*) and fructose-bisphosphate aldolase (*fba*). Members of this phylum also lacked genes for TCA cycle and electron transport chain as well as most of the sugar utilization genes.

Bacteroidota genomes followed by Verrucomicrobiota, Firmucutes_A and Spirochaetes showed the highest capability for metabolizing carbohydrate polysaccharides. They can utilize a wide range of carbohydrate monomers, including rhamnose, mannose, xylose, fructose, galactose, arabinose, fucose and galacturonic acid (Fig. 5). In certain taxa, including members of Alphaproteobacteria, Patescibacteria and Cyanobacteria, no hit corresponding to the key isomerase or kinase enzymes required for sugar metabolism was detected, suggesting their lack of sugar utilization and their dependence on other rumen microorganisms. Since most genomes were incomplete, failure to detect key metabolic genes could be artifactual. In contrast to Alphaproteobacteria, Gamaproteobacteria genomes, however, showed a wide carbohydrate utilization capacity and the ability to ferment VFAs, such as butyrate, acetate, succinate and formate. The reconstructed Alphaproteobacteria genomes belonged to the orders Rs-D84 (RUI088, RUI239 and RUI567) and UBA3830 (RUI108, RUI449 and RUI463). Members of the order UBA3830 were also detected in co-assembly of rumen metagenome sequences of cow, goat, reindeer and red deer^43^. In addition to their low carbohydrate utilization, Alphaproteobacteria also showed minimal carbohydrate degrading and fermentation capacity with only members of Rs-D84 to be capable of lactate and acetate fermentation, while we failed to detect the key enzymes for fermentation metabolism in UBA3830 genomes. Alphaproteobacteria also lacked TCA cycle enzymes, which further supported their fermentative lifestyle and their potential contribution to VFA fermentation in the camel rumen.

Out of six Verrucomicrobiota RUIs, three were assigned to the class Kiritimatiellae (RUI187, RUI347 and RUI391), two to Lentisphaeria (RUI186 and RUI478) and the remaining one to Verrucomicrobiae (RUI087). Members of this phylum showed widespread lignocellulose-degrading activity and sugar-utilizing capacity and are therefore thought to make a significant contribution to rumen function. The absence of laccase demonstrated their limited contribution to lignin breakdown. They also appeared to engage in acetate, butyrate and propionate metabolism.

Within the Firmicutes_C phylum, seven RUIs genomes were reconstructed and all of them were associated with the family Selenomonadaceae. At the genus level, two were associated with the genus Selenomonas (RUI343 and RUI207), two with Schwartzia (RUI215 and RUI279), one with Anaerovibrio (RUI081) and one as UBA7018 (RUI390), while the last one remained unassigned at the genus level (RUI020). These genomes displayed the potential for malate utilization and propionate fermentation. They also showed limited carbohydrate degrading potential with only starch and glucans as preferred substrates. Thirty-eight RUIs were assigned to the Spirochaetota phylum, of which 34 belonged to the family Treponemataceae and four to Sphaerochaetaceae. Members of this phylum showed broad lignocellulose-degrading activity as well as sugar utilizing and fermentative capacities, corroborating with their significant roles in the rumen function. The unique feature of these bacterial clades seemed to be their inability to metabolize fucose, an observation which is also reproduced in Spirochaetota genomes identified in cattle’s rumen^19^.

Out of the five Patescibacteria RUIs, four were associated to the class Saccharimonadia (RUI278, RUI350, RUI400 and RUI495) and one to the class Gracilibacteria (RUI200). Patescibacteria are obligatory fermentative bacteria which depend on both lactate and acetate fermentation^44^. We noted evidence for both lactate and acetate fermentation (*ldh* and *ackA*) in Saccharimonadia while Gracilibacteria genomes lacked both enzymes. Failure to detect fermentation-related genes in Gracilibacteria genome could be artifactual owing to its incomplete genome (~78%). The characteristic feature of Patescibacteria genomes was the absence of PFK which catalyzes the conversion of fructose 6-phosphate into fructose-1,6-bisphosphate, a key step in the glycolysis pathway. In the absence of this enzyme, the glycolysis pathway is thought to be completed through a metabolic shunt, including the non-oxidative PPP^45^. An interesting finding was the detection of enolase in these genomes, which was considered as an almost missing enzyme in this clade^46,47^, suggesting the existence of extensive metabolic diversification among the members of this phylum inhabiting different ecological niches.

Patescibacteria also showed limited carbohydrate degrading capacity with only a single copy of α-amylase (GH97, GH57 and GH13_8), glucosidase (GH3, GH_13_30 and GH13_31), cellobiohydrolase (GH6), lysozyme (GH23 and GH25) and polygalacturonase (GH28 and GH114) along with acetyl xylan esterase (CE4 and CE5) and pectin acetylesterase (GH13) found in certain Saccharimonadia genomes. No CAZyme encoding gene was detected in the Gracilibacteria genome. Patescibacteria also encode for type IV pili, structures supporting their attachment to other microbes.

### Identification and annotation of biosynthetic gene clusters (BGCs) in the RUIs

The reconstructed RUIs were screened for secondary metabolite biosynthetic gene clusters (BGCs) using AntiSMASH^48^. We identified 1003 putative BGCs in 425 RUIs belonging to 18 different phyla (Supplementary Data 8), indicating that camel rumen microbes are valuable resources for secondary metabolite mining. The predicted BGCs belonged to 19 different families with the most abundant ones (frequencies >1%) to be non-ribosomal peptide synthetases (NRPSs, *n* = 427), followed by sactipeptides (*n* = 152), arylpolyenes (*n* = 147), bacteriocins (*n* = 97), betalactones (*n* = 80), terpenes (*n* = 24), lassopeptides (*n* = 13), proteusins (*n* = 10) and lanthipeptides (*n* = 10). More than 62% of the BGCs were found in Firmicutes_A, 15% in Bacteriodota, 6% in Spirochaetota and less than 5% in Firmicutes (i.e., Bacilli). Analysis of the distribution of BGC families in different phyla revealed a relatively high abundance of NRPSs in Firmicutes and Desulfuromonadota. Bacteriodota along with Verrucomicrobiota and Spirochaetota contained numerous arylpolyene clusters, which were rarely detected in Firmicutes. Bacteriocin encoding BGCs were exclusively detected in Firmicutes, Spirochaetota and Actinobacteriota. Ribosomally synthesized and post-translationally modified peptides (RiPPs), including sactipeptides, lanthipeptides, lassopeptides, and proteusins were only detected in Gram-positive bacteria of the Firmicutes phylum.

## Discussion

### Rumen microbiota shows redundancy in substrate colonization

Camels naturally feed on recalcitrant forages and thus their gastrointestinal microbiota must have been adapted to breakdown complex lignocellulosic substrates. Here we showed that rumen microbiota of camel display redundancy in colonization of forages of varying physicochemical properties highlighting the specific adaptation mechanisms that enable them to utilize different plant-based substrates. Redundancy in substrate utilization is largely fueled by the high taxonomic and functional diversity of the rumen microbiota. Redundancy is a key feature of rumen microbiota that is characterized by the existence of same metabolic capabilities across different taxonomic lineages^49^. The ability to colonize different plant-based substrates proved to be an important aspect of rumen microbiota that enables the host to harvest energy from diverse plant-based nutrients. This is fundamentally important for animals such as dromedary camels that live in deserts and are forced to feed on dogged lignocellulosic plants. Similar findings have been reported in rumen microbiome of cattle as they also showed the ability to colonize substrates with different level of digestibility^13^. This ability may also grant their maintenance in the dynamic environment of rumen and thus prevent their washout along rumen fluids^10^.

It has been shown that rumen microbiota, particularly cellulose degraders, compete for colonization and attachment to cellulosic substrates^50,51^. Lignocellulose-degrading bacteria employ different mechanisms for fiber adhesion and degradation and thus their lignocellulose-degrading capabilities may vary on different lignocellulosic substrates, a phenomenon that also affects fermentation metabolism in the rumen^52^. This is also reflected in their colonization behavior as rumen cellulolytic bacteria tend to colonize different parts of plant substrate, depending on their lignocellulose content^53^. Studies have shown that rumen microbiome composition is heavily influenced by diet^24^, however, any diet-induced changes in the rumen community can be restored. This highlights another critical feature of rumen microbiome, its resiliency, that is the capacity to restore to its original state after any internal or external perturbations^49^. Rumen microbiome resiliency could be linked to their ability to colonize different plant-based substrates, which grants their maintenance even under less-desirable substrates.

### Carbohydrate degradation and fermentation metabolism are distributed in trophic-like levels

Metabolic characterization revealed significant diversity, with only certain taxonomic clades able to fully metabolize sugar monomers or possess full fermentative capacity. Redundancy in functional capabilities across biofilm-forming microbes is also evident in our rumen metagenome data (Fig. 5). Functional redundancy is fundamentally important since it enables the rumen microbiome to maintain a stable metabolic function under constant influence of diet, host and other environmental factors that may affect community composition. This has been experimentally demonstrated in bovine rumen microbiome, where animals with different rumen community compositions exhibited almost similar fermentation characteristics^54^. We found that carbohydrate degradation and sugar utilization capabilities were often distributed across limited number of rumen microbiome lineages which are among abundant members (mainly Clostridia and Bacteroidia) of the community. This suggested that other members of the rumen community might be dependent on these microbes for survival in the rumen. These interdependencies are likely key to the rumen microbiome function as they greatly enhance synergistic interactions between microbes during lignocellulose degradation in fluctuating environment of the rumen. Metabolite auxotrophies are known to strengthen cooperation among members of a microbial community and thus promote stabilization of the whole community^55^. In rumen microenvironment, these cross-metabolite tunneling create trophic-like levels across rumen microbiota as a specific mechanism to improve metabolism in the rumen^5,56^. This is also in agreement with the notion that every rumen microbiome species fills a specific niche in the rumen (niche partitioning) and thus all the members of rumen community are functionally important for proper rumen function. Only certain fiber colonizing bacteria occupied the first trophic-like level (level I) in the camel rumen microbiome metabolic network. These included fiber-degrading classes of Clostridia, Bacteroidia, Fibrobacteria, Bacilli, Spirochaeta, Lentisphaeria, Kritimatiellae, Planctomycetes and Negativicutes which have been equipped with diverse enzymatic systems for degradation of polysaccharides into soluble oligos or sugar monomers (Fig. 5A). Particularly, Clostridia, Bacteroidia and Spirochaeta encoded for ~90% of all glycoside hydrolases in the fiber-adhering community of the camel rumen and thus thought to play central role in polysaccharide degradation in the rumen. Microbes in this trophic-like level also varied with respect to their content for GHs. The highest GH densities were recorded for members of Verrucomicrobiota, Bacteroidota, Fibrobacterota, Planctomycetota and Spirochaetota. Due to their uncultured nature, the role of Verrucomicrobiota in the rumen function had been neglected for years but culture-independent metagenomic approaches have now shed light on their unique role in lignocellulose degradation in the rumen. Verrucomicrobiota are known to encode for a wide range of carbohydrate degrading enzymes, peptidases and sulfatases and thus thought to be well-adapted for lignocellulose degradation in the rumen^57^. Within this phylum, members of the classes Lentisphaeria and Kiritimatiellae showed wide carbohydrate polymer and sugar utilization capacities while those of Verrucomicrobiae had a limited capacity and thus may have a more specific role in the rumen function. Similarly, Spirochaetota also showed broad carbohydrate degrading and sugar metabolizing function corroborating their key roles in the rumen function. Previous metagenome sequencing of rumen microbiota of browsing camels also revealed significant potential of Spirochaetota for contributing to cellulose and hemicellulose-degrading enzymes^30^. The association of Spirochaetota, particularly members of the genus *Treponema* with pectin degradation in the rumen^58^ and xylan degradation in termite hindgut^59^ has already been demonstrated. The uneven distribution of CAZymes across microbes constituting this trophic-like level further highlight the logic behind the necessity of cooperation of multiple different lignocellulose-degrading lineages for efficient and timely digestion of fiber in the rumen^60^.

Although most microbes in this trophic level capable of metabolizing fiber-degradation products but the leakage or excretion of energy-accessible metabolites supports the growth of other microbes that are unable to breakdown complex polysaccharides^56^. These microbes constitute trophic-like level II which are capable of utilizing sugars or metabolites released during polysaccharide degradation by microbes in trophic level I. This is functionally important as fiber-degrading microbes dependent on certain metabolites and precursors generated by microbes in this trophic level for their survival and multiplication (e.g. the biosynthesis of amino acid and fatty acids)^61^. Although these microbes are unable to degrade fiber, their dependance to degradation or fermentation products of fiber-degrading bacteria necessitates their close interaction, likely by co-attachment to feed particulates^60^.

In the last trophic level (level III), some of the products of metabolism in the first two trophic levels are further metabolized into SCFA metabolites such as acetate, propionate, and butyrate which are favored metabolites for the host animal. Members of this trophic level are important for the host animal nutrition and their community is thought to be less complex in animals with higher nutrient use efficiency^6^. In fiber-attaching community of camel rumen microbiome acetate and butyrate metabolism are widespread but the propionate is less frequently occurred. Methane metabolism is also located in this trophic-like level which is performed by methanogenic archaea. Methanogens utilize hydrogen generated in the last two trophic-like levels (II and III) to reduce carbon dioxide to methane^62^. Members of Bacteroides and Clostridia including species of Ruminococcus, Clostridium, and Butyrivibrio are the main source of fermentative hydrogen production in the rumen^63,64^. Methane serves as an electron-sink and its production is required to maintain a low hydrogen partial pressure in the rumen, which in turn essential for the optimal biomass hydrolysis^65^. Only single archaea genome bin (RUI021) could be reconstituted which was identified to be a member of Methanobrevibacter genus. The low representation of archaea genomes might be due to their inability to attach to lignocellulosic substrates.

### Lactate fermentation is not widespread across the camel rumen microbiota

The camel rumen’s microbiota showed limited lactate fermentation metabolism as only certain members of Clostridia, Bacilli, Spirochaetia, and Proteobacteria scored positive for lactate fermentation. The potential for the conversion of lactate to propionate was also rarely detected in certain members of Firmicutes phylum. It has been shown that lactate metabolism is linked to a low methane emission since both lactate utilization and methanogenesis compete for hydrogen in the rumen^66^. On the other hands, the absence of lactate fermentation could be beneficial for the host animal, since excess lactate fermentation, particularly under non-fibrous/starch-rich diet (cereal grain or concentrate), causes ruminal acidosis, a digestive disorder characterized by excess lactate accumulation and a sudden drop in ruminal and blood pH^67^. This could be considered as a potential side effect of ruminal microbial fermentation for the host animal, which could be mitigated by microbiome engineering approaches. For example, administration of lactate utilizing bacteria, such as *Megasphaera elsdenii*, has been shown to ameliorate rumen acidosis^68^. Species, such as *Selenomonas ruminantium*, are also known as lactate consuming bacteria that are able to convert lactate to pyruvate and thus play a key role in the lactate utilization and the reduction of methane production in the rumen^5,69^. Lactate fermenters are known to increase in abundance under condition of rapid sugar fermentation and thus their low representation in camel rumen might be due to the recalcitrant nature and low sugar content of plant materials constituting camel’s diet.

### The abundance of Patescibacteria is relatively high in the camel rumen

We found a high representation and even distribution of Patescibacteria, formerly known as Candidate Phyla Radiation (CPR), in microbial communities associated with different substrates. Patescibacteria are uncultured bacteria that have lost genes for major metabolic pathways such as de novo biosynthesis of amino acids, nucleotides, fatty acids and cofactors, and thus they depend on other microorganisms for their survival^45,46^. Members of this phylum are thus typical examples of rumen microbiota which have been evolved an auxotrophic metabolism. They have evolved pili-like structures which enable them to attach to other microbes^70^.These structures may act as tunnels for the exchange of metabolites thus facilitating the direct import of metabolites from their syntrophic partner. With no major lignocellulose degrading and sugar fermenting potential, the existence of these bacteria among fiber-attached microbiota of camel rumen suggested that their attachment to other polysaccharide degrading and biofilm-forming bacteria granted their recovery from metagenome sequences. This is an example of how physical interactions or co-attachments to solid substrates facilitate metabolic interactions among microbes occupying different trophic levels. Further research would be required to elucidate the exact function of these bacteria in the rumen.

### Biological gene clusters are widely distributed across rumen microbiota

Exploration of camel rumen microbiota for microbial secondary metabolites revealed a relatively high abundance and diversity of their encoding gene clusters (BGCs). BGCs are clusters of two or more genes encoding enzymes or enzyme complexes responsible for the biosynthesis of specific secondary metabolites, many of which are small molecules with pharmaceutical and agricultural importance, such as antibiotics (penicillin and erythromycin), immunosuppressants (cyclosporins) and drugs (statins)^71,72^. NRPS and Sactipeptides are among abundantly detected BGCs in the camel rumen. As the most abundant and structurally diverse classes of BGCs, NRPSs are thought to play a key role in microbial interaction. Given their high abundance and their presence in microbes of different taxonomic origins, they appeared to have an important function in the camel rumen. Sactipeptides, on the other hand, have a narrow spectrum antimicrobial activity against Clostridia, rendering them attractive for antibiotic development. Recently, the sactipeptide ruminococcin C was isolated and characterized from *Ruminococcus gnavus* in the human gut microbiome, which showed potent activity against human pathogen *Clostridium perfringens*^73^. As ribosomally synthesized and post-translationally modified peptides (RiPPs), lanthipeptides contain thioether cross-linked amino acids, lanthionines, in their structure^74^. They display a wide range of biological activities, including antimicrobial, antinociceptive and antiallodynic functions, making them putative candidates for therapeutic applications^75^. Another group of RiPP secondary metabolites identified in Firmicutes were lassopeptides which are known for their antimicrobial activities, by targeting other microbes they likely give the host bacteria a competitive advantage under nutrient limitation^76^. Bacteriocins constitute a large and diverse group of ribosomally synthesized peptides produced by both bacteria and archaea with potential antimicrobial activity against bacteria closely related to the producer strain^77^. For example, certain cellulolytic members of rumen community produce bacteriocin-like peptides to inhibit the growth of their competitors^78,79^. Due to widespread functional redundancy in rumen microbiota, there is huge potential competition between members of rumen community, specifically between those co-occupy a unique ecological niche^60^. In vitro co-culture studies have reported negative interactions between major cellulose-degrading bacteria, including *R. albus*, *R. flavefaciens* and *F. succinogenes* which could be mechanistically linked to BGCs^50^. Recent discovery of 14,814 BGCs in 8160 rumen MAGs from different ruminant species revealed the high diversity and abundance of secondary metabolites in rumen microbiomes, implying their potentially important functions in the rumen^80^. This finding suggests a key role for these small molecules in regulating the structure and function of rumen microbiota. They may also function to protect host from invading pathogens. Our analyses also provide accumulating evidence that the rumen microbiomes are an untapped source of natural bioproducts with broad pharmaceutical properties.

## Conclusion

This study provides the most comprehensive set of metagenome-assembled genomes from the camel rumen along with a deep characterization of their taxonomic diversity and functional significance for the host animal. The majority of the reconstructed RUIs (> 80%) were estimated to be novel with no well-characterized representative among reference genomes or previously assembled rumen MAGs. This potential diversity is likely driven by geographically distributed species/strains, host species variations and differences in dietary forages and their associated epiphytic microbial communities. A deeper analysis of the genomic potential of the reconstructed genomes for CAZymes and the associated pathways for fermentation and utilization of the degraded products revealed a significant diversity among different members of the rumen community and their widespread interdependencies for intermediate metabolites. This finding suggests that rumen microbial communities have been evolutionarily selected to complement each other and to support the host animal with energy-rich fermentation products, intermediate metabolites and vitamins. The existence of a wide range of secondary metabolite gene clusters with potentially diverse antimicrobial properties highlights the key role of these small metabolites in rumen microbial interaction and function. Particularly, these naturally occurring antimicrobial products likely contribute to shaping the structure of the microbial community in the gut environment by targeting other microbes, especially pathogens.

## Methods

### Ethical statement

The animal experiment was approved by the Ethics Committee for Animal Experiments of the Animal Science Research Institute of Iran.

### Sample preparation and rumen incubation

Two single-humped camels aged 3–4 years were isolated from a naturally grazing camel herd and reared in a barn for at least 2 months. Animals were fed daily with a mixture of alfalfa hay (30 %) and wheat straw (70 %) and had free access to drinking water. They were held off feed and water for at least 12 h before rumen cannulation. Rumen cannulation surgery was performed according to the American College of Veterinary Surgeons using a two-stage rumen cannulation technique^81^ by a qualified veterinary surgeon. After fistulation, the surgical site was cleaned daily, and the cannula’s outer surface was treated with antiseptic solution for at least 7 days. Animals were cared for at least 2 months before feed incubation in the rumen.

Eleven different lignocellulosic substrates with varying physicochemical properties, including straws (both stems and leaves) of wheat (WS), rice (RS), corn (CS), common reed (CR), koshia (KS), sorghum (SG), salicornia (SC), camelthorn (AP), leaves of date palm (DP), and pulps of sugar beet (SS) and sugarcane bagasse (SB) were selected for rumen incubation. Most of these lignocellulosic materials are used as forage and/or supplements for feeding to domestic ruminants in Iran and many other countries. Forage physicochemical properties, including dry matter (DM), neutral detergent fiber (NDF), acid detergent fiber (ADF) and acid detergent lignin (ADL) were measured using the detergent fiber system^82^.

Samples were collected during September 2018. Plant materials were cut into pieces of ∼2 mm in length and 5 ± 0.05 g of each forage material was weighed and placed into heat-sealed nylon bags (5 × 10 cm; 50 μm pore size). For each biomass, four bags were placed into the rumen of each fistulated animal shortly after the morning meal. Since the number of bags was too high for simultaneous incubation in the rumen, they were placed consecutively in two random batches (one with six forages and another with five forages). Animals were fed a ration of 70% wheat straw and 30% concentrate and had free access to drinking water during the incubation. One bag per substrate was retrieved from each animal after 24, 48, 72, and 96 h of rumen incubation. After a brief washing of the retrieved bags with distilled water, the contents were transferred to sterile falcon tubes and subjected to three additional rounds of washing to ensure liquid-born and loosely attached microbiota were discarded. Plant materials were then treated with dissociation buffer consisting of 0.1% (v/v) Tween 80, 1% (v/v) methanol and 1% (v/v) tertiary butanol (adjusted to pH 2) for 15 min to recover microbial cells firmly attached to feed particles. The samples were vigorously vortexed every 1 min to speed up the detachment of microbial cells. Plant solid residues were sedimented by a centrifugation at 500 × *g* for 5 min and the liquid supernatant containing microbial cells was transferred to a new sterile container. These steps were repeated at least three times and the collected liquids were finally centrifuged at 12,000 × *g* for 10 min to sediment the detached microbial cells. The collected microbial cells were stored at −80 °C until DNA extraction.

### DNA extraction and metagenome sequencing

Total microbial genomic DNA was extracted using the QIAamp® DNA Stool Mini Kit (Qiagen, Hilden, Germany). The quality and quantity of extracted DNA were evaluated using a NanoDrop ND-1000 Spectrophotometer (Thermo Fisher Scientific Inc., Wilmington, DE, USA) and agarose gel electrophoresis. Metagenome library preparation was conducted using a TrueSeq DNA Sample Prep Kit (Illumina, San Diego, CA, USA) according to the manufacturer’s instruction. The quantity of each library was measured fluorometrically using the Qubit 3.0 Fluorometer (Invitrogen, Carlsbad, CA, USA). Metagenome sequencing was performed at Novogene Inc. (Nanjing, China) using an Illumina NovaSeq 6000 Sequencing System with 150 bp paired-end sequencing of ~350 bp insert fragment.

### Taxonomic composition of whole metagenome sequences

Raw metagenome sequences were used to infer the taxonomic composition of the whole microbiome using MetaPhlAn v3.0.7^83^, which uses 1.1 million unique clade-specific marker genes from ~100,000 reference genomes for accurate and unambiguous taxonomic classification and abundance estimation of microbes in a metagenome. The abundance table was used to infer beta diversity of microbiota across different substrates based on UniFrac and Bray_Curtis dissimilarity matrices.

### Assembly of metagenome sequences and reconstruction of genome bins

Quality filtered metagenome sequences were de novo assembled using MEGAHIT v1.2.9^84^ with the parameters -k-min 31, -k-max 141, -k-step 10, -min-count 2 and -min-contig-len 200. Total sequences obtained from microbial cells attached to all lignocellulosic substrates were co-assembled to increase the chance of recovering contigs from low abundant members of the rumen microbiome. The coverage of contigs (>2000 bp) was estimated by mapping reads to the contigs using Bowtie2 v2.3.5 with default parameters^85^. The resulting alignment files (SAM format) were converted into BAM files and sorted based on sequence coordinates using Samtools v1.9^86^. Contigs were binned into rumen uncultured isolates (RUIs) based on their coverages across the samples and their tetranucleotide frequencies using MetaBat2 v2.14 with the following options: –minContigLength 2000 and –minContigDepth 2^87^. The RUIs were quality filtered and dereplicated using dRep v2.3.2^88^. Completeness and contamination of the RUIs were assessed based on the presence/absence or duplication of universally conserved protein-coding marker genes using CheckM v1.0.18^89^. Only RUIs (*n* = 591) with completeness ≥75% and contamination ≤10% were selected for further analysis.

### Taxonomy and functional annotation of the RUIs

Taxonomies were assigned to the RUIs using GTDB-Tk v0.3.3^26^. The tree showing phylogenetic relationships of the RUIs was constructed using PhyloPhlAn v0.97^90^, visualized using bioconductor package ggtree^91^ and annotated based on phylum level taxonomies inferred by GTDB-Tk. RUIs were compared based on average nucleotide identity (ANI) to the cultured representative genomes from cow rumen n = 408^28^, the set of rumen metagenome-assembled genomes (MAGs) from the cow rumen *n* = 4941^29^, Taleshi cattle rumen *n* = 538^19^, and camel rumen *n* = 65^30^ using FastANI v1.1 (https://github.com/ParBLiSS/FastANI) with the option –fragLen 2000. tRNAs were predicted using tRNAscan-SE v2.0.6^92^. Genes encoding ribosomal RNAs (rRNAs) were identified using barrnap v.0.9 (https://github.com/tseemann/barrnap).

### Differential composition and abundance of the RUIs

To estimate the abundance of the RUIs, reads were aligned to contigs larger than 2 kb. The abundance of genome bins was then estimated using the quan_bins module from MetaWRAP pipeline^33^. The differential abundance of genome bins at different taxonomic levels was assessed by ANCOM^93^ considering the absolute abundance of bins across samples. A heatmap representing the abundance of genome bins was constructed using average linkage method and Spearman correlation distance matrix.

For community diversity analysis, the abundance table along with the taxonomies inferred by GTDB-Tk were converted into a json-formatted biom file. Community differences between substrates and incubation times were assessed using permutational multivariate analysis of variance (PERMANOVA) following the Adonis function in R package vegan v2.5-5^94^ based on weighted and unweighted UniFrac distance matrices. To test for homogeneity of dispersions, permutational multivariate analysis of dispersion (PERMDISP) was performed using the beta-disper function in vegan. The abundance profiles of bins (summarized at taxonomic level of phylum) were further correlated with forage physicochemical properties (i.e., NDF, ADF and ADL) as explanatory factors using redundancy analysis (RDA). Specific correlations were inferred using rcorr function of Hmisc R package. Significant differences were tested using analysis of variance (ANOVA) in PAST statistical software^95^.

### Functional annotation of the whole metagenomes

Protein-coding genes in contigs larger than 200 bp were predicted using Prodigal v2.6.3 in metagenome mode^96^. Open reading frames (ORFs) larger than 50 amino acid (aa) residues were BLASTP searched against NCBI nr (November 2019) and env_nr (April 2021) databases using DIAMOND v2.0.5.143^97^ with an e-value cutoff 1e-5 and in -sensitive mode. Kyoto Encyclopedia of Genes and Genomes (KEGG) orthologs were identified by searching ORFs ≥ 50 aa against the KofamKOALA database of profile Hidden Marcov Models using KofamScan v1.3.0^98^. Clusters of Orthologous Groups (COG) annotation^99^ was performed using DIAMOND.

### Identification and annotation of carbohydrate active enzymes (CAZymes)

To identify CAZymes, predicted proteins were searched against dbCAN database v6 using run_dbcan v2.0.11^100^, considering a minimum query coverage of 35% and an e-value cutoff 1e−15. Polysaccharide utilization loci (PULs) were predicted using PULpy^101^ by applying the same criteria. Amino acid sequences of the predicted CAZymes were BLASTP searched against the NCBI nr database and dbCAN database using an e-value cutoff at 1e−30 and a maximum target sequence of 1. Signal peptides in the predicted proteins were identified using SignalP v4.1 operating in both Gram-positive and Gram-negative modes^102^. The abundance of CAZymes were determined by aligning reads to their ORFs using Salmon^103^. Genes with significant differences in abundance were identified using normalized transcript per million mapped reads (TPM) in R package DESeq2. Bonferroni-corrected P-values less than 0.01 were considered to be statistically significant.

### Metabolic annotation of the RUIs

The predicted proteins were searched for KEGG orthologs using KofamScan as described above. The KEGG orthologs for the key enzymes of major metabolic pathways, including glycolysis, pentose phosphate (PPP), the tricarboxylic acid (TCA) cycle and fermentation of short chain fatty acids (SCFAs), were retrieved from the KofamScan output. The ability to metabolize monomeric carbohydrates was determined by searching for enzymes encoding glucose-6-phosphate isomerase (EC:5.3.1.9, K01810), fructokinase (EC:2.7.1.4, K00847), galactokinase (EC:2.7.1.6, K00849), L-arabinose isomerase (EC:5.3.1.4, K01804), L-fucose/D-arabinose isomerase (EC:5.3.1.25 5.3.1.3, K01818), xylose isomerase (EC:5.3.1.5, K01805), mannose-6-phosphate isomerase (EC:5.3.1.8, K01809) and L-rhamnose isomerase (EC:5.3.1.14, K01813).

The ability to metabolize carbohydrate monomers into SCFAs was also determined for RUIs. Butyrate production was confirmed by the presence of genes for phosphate butyryltransferase (*ptb*), butyrate kinase (*buk*) and butyryl-CoA:acetate CoA-transferase (*but*). Lactate dehydrogenase (*ldh*) was used as a marker of lactate production. The presence of phosphate acetyltransferase (*pta*) and acetate kinase (*ackA*) genes proved the ability to ferment acetate. Malic enzyme (*men*), fumarate hydratase (*fum*) and fumarate reductase (*frd*) were searched as key enzymes of succinate production. Propionate production was validated by the presence of three key enzymes of methylmalonyl-CoA decarboxylase (*mmdA*), methylmalonyl-CoA mutase (*mutA*) and methylmalonyl-CoA epimerase (*mce*). The potential for degradation of carbohydrate polymers was also evaluated by looking for enzymes involved in the degradation of lignocellulosic polymers, including cellulose, xylan, xyloglucan, chitin, pectin and starch. Details of enzyme families used for this analysis are presented in Supplementary Data 3c.

Identification and annotation of biological gene clusters (BGCs) was carried out using AntiSMASH v5.1.2^48^. BGC regions were defined based on their locations in contigs.

### Reporting summary

Further information on research design is available in the Nature Research Reporting Summary linked to this article.

## Supplementary information

Supplementary figures

Supplementary Data 1

Supplementary Data 2

Supplementary Data 3

Supplementary Data 4

Supplementary Data 5

Supplementary Data 6

Supplementary Data 7

Supplementary Data 8

Reporting Summary Checklist

## Data Availability

Raw sequence data have been deposited in the NCBI Short Read Archive (SRA) under BioProject ID PRJNA746430. The sequences of contigs and RUIs have also been deposited under the same BioProject ID.
