# Supplementary material for: Functional and phylogenetic analyses of camel rumen microbiota associated with different lignocellulosic substrates
Source: NPJ Biofilms Microbiomes. 2022 Jun 8;8:46. doi: 10.1038/s41522-022-00309-9 (PMC9177762; doi:10.1038/s41522-022-00309-9)
Supplement: Supplementary file 1 — Supplementary figures [file 41522_2022_309_MOESM1_ESM.pdf]

Functional and phylogenetic analyses of camel rumen microbiota associated to different lignocellulosic substrates

Javad Gharechahi[1#], Sajjad Sarikhan[2#], Jian-Lin Han[3,4], Xue-Zhi Ding[5*] and Ghasem Hosseini Salekdeh[2,6]*

1. Human Genetics Research Center, Baqiyatallah University of Medical Sciences, Tehran, Iran
2. Department of Systems Biology, Agricultural Biotechnology Research Institute of Iran, Agricultural Research, Education, and Extension Organization, Karaj, Iran
3. Livestock Genetics Program, International Livestock Research, Institute (ILRI), 00100 Nairobi, Kenya
4. CAAS-ILRI Joint Laboratory on Livestock and Forage Genetic Resources, Institute of Animal Science, Chinese Academy of Agricultural Sciences (CAAS), 100193 Beijing, China
5. Key Laboratory of Yak Breeding Engineering, Lanzhou Institute of Husbandry and Pharmaceutical Sciences, Chinese Academy of Agricultural Sciences (CAAS), 730050 Lanzhou, China
6. Department of Molecular Sciences, Macquarie University, North Ryde, NSW, Australia

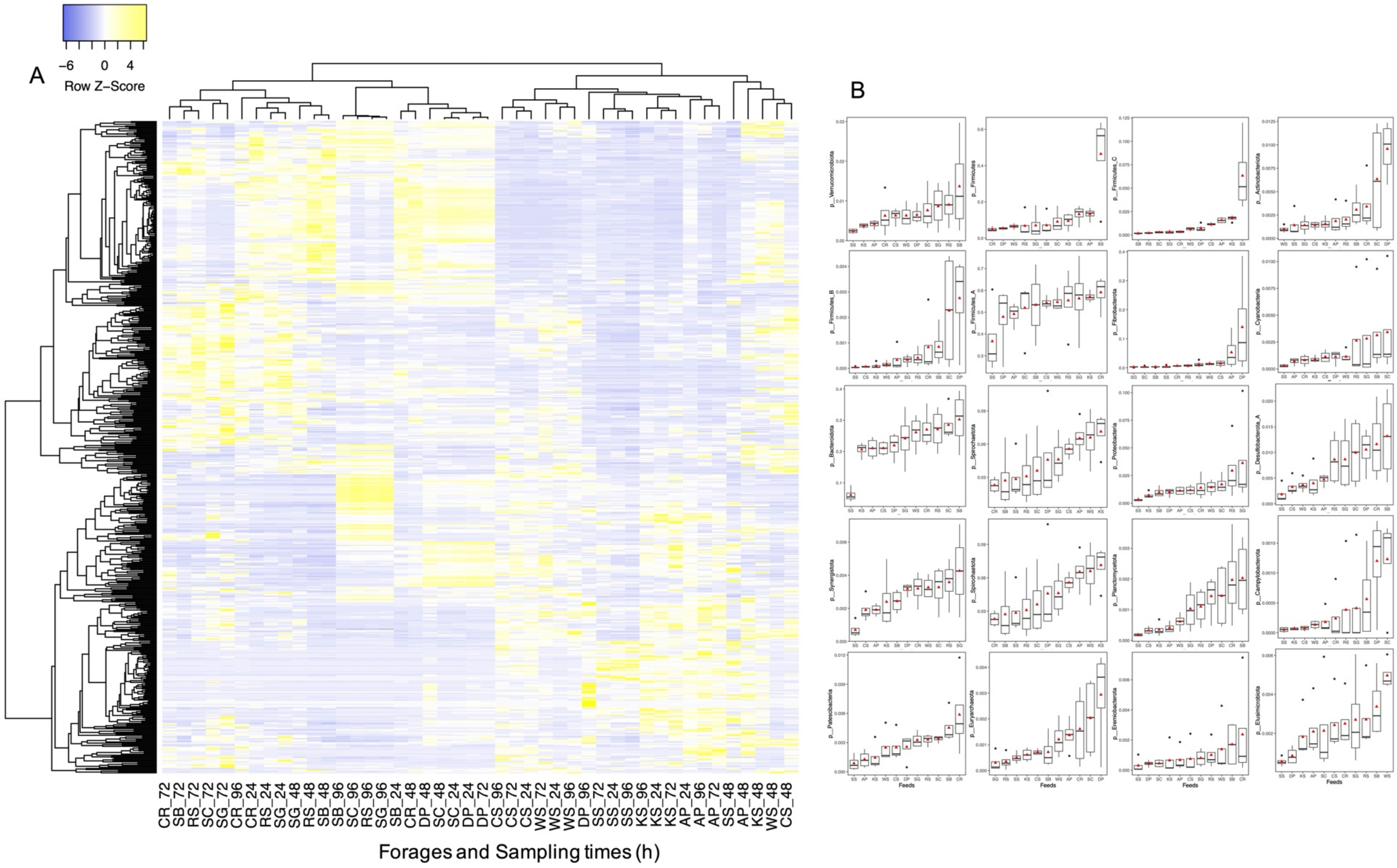
A
B
−6 0 4
Row Z−Score
CR_72 SB_72 RS_72 SC_72 SG_72 CR_96 CR_24 RS_24 SG_24 SG_48 RS_48 SB_48 SB_96 SC_96 RS_96 SG_96 SB_24 CR_48 DP_48 SC_48 SC_24 DP_24 DP_72 CS_96 CS_72 CS_24 WS_72 WS_24 WS_96 DP_96 SS_72 SS_24 SS_96 KS_96 KS_24 KS_72 AP_24 AP_96 AP_72 SS_48 AP_48 KS_48 WS_48 CS_48
Forages and Sampling times (h)
Feeds

Fig. S1: The abundance distribution and composition of RUIs attached to the 11 substrates incubated for 24, 48, 72 and 96 h in the camel rumen. (A) Heatmap illustrates the abundance profile of 591 RUIs across 44 samples. The RUIs were hierarchically clustered based on their abundances estimated by mapping reads to the contigs larger than 2 Kbp using an average linkage method and Spearman-rank correlation distance measurement implemented in Heatmapper [104]. (B) box plots show the abundance profile of the major phyla in the incubated feeds. The relative abundances of RUIs were summarized at phylum level according to the taxonomies inferred by GTDB-Tk. The boxes represent the median (center line) and interquartile ranges, red triangle represents mean and whiskers extend to one and a half times of the interquartile range.

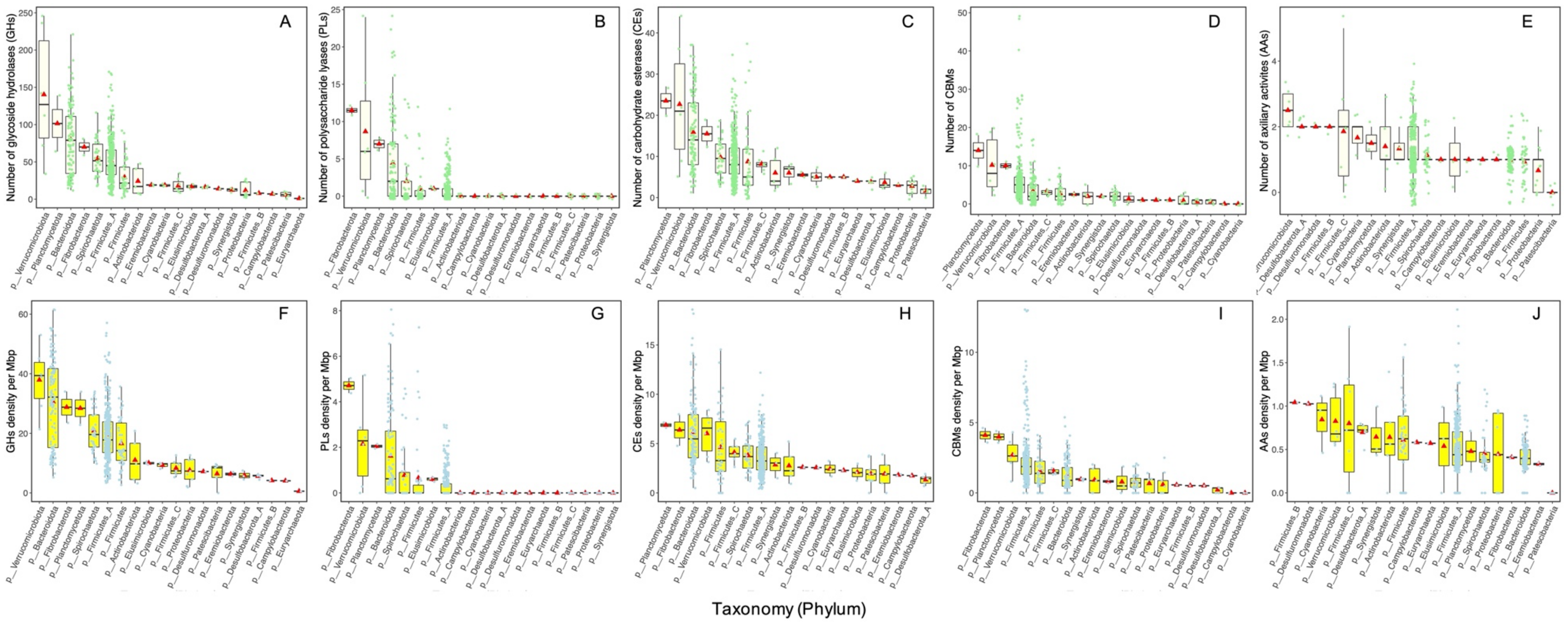

Fig. S2: Number of CAZymes (A-E) and their average densities per Mb of genome size (F-J) in RUIs. The RUIs were classified at phylum level based on taxonomies inferred by GTDB-Tk. Taxa were sorted according to their average CAZyme count or CAZyme density. The boxes represent the median (center line) and interquartile ranges, red triangle represents mean and whiskers extend to one and a half times of the interquartile range.
